# Longitudinal visceral muscles in *Drosophila* fully dedifferentiate and fragment prior to their reestablishment during metamorphosis

**DOI:** 10.17912/micropub.biology.000756

**Published:** 2023-03-15

**Authors:** Dorothea Schultheis, Manfred Frasch

**Affiliations:** 1 Institute of Neuropathology, Universitätsklinikum Erlangen, University of Erlangen-Nuremberg, Erlangen, Bavaria, Germany; 2 Division of Developmental Biology, Department of Biology, University of Erlangen-Nuremberg, Erlangen, Bavaria, Germany

## Abstract

Although the *Drosophila* longitudinal visceral muscles have been shown to undergo major morphological changes during the transition from larval to adult gut musculature, there have been conflicting views as to whether these muscles persist as such during metamorphosis or whether they are built anew (Klapper 2000; Aghajanian et al. 2016). Here we present our independent analysis using *HLH54Fb-eGFP* as a cell type specific marker, which strengthens the proposition by Aghajanian et al. (2016) that the syncytial larval longitudinal gut muscles completely dedifferentiate and fragment into mononucleated myoblasts during pupariation before they fuse again and redifferentiate to form the adult longitudinal gut muscles.

**Figure 1. Midgut preparations from HLH54Fb-eGFP line stained for GFP, F-actin, and nuclei, showing a time course of dedifferentiation and redifferentiation events during metamorphosis:**
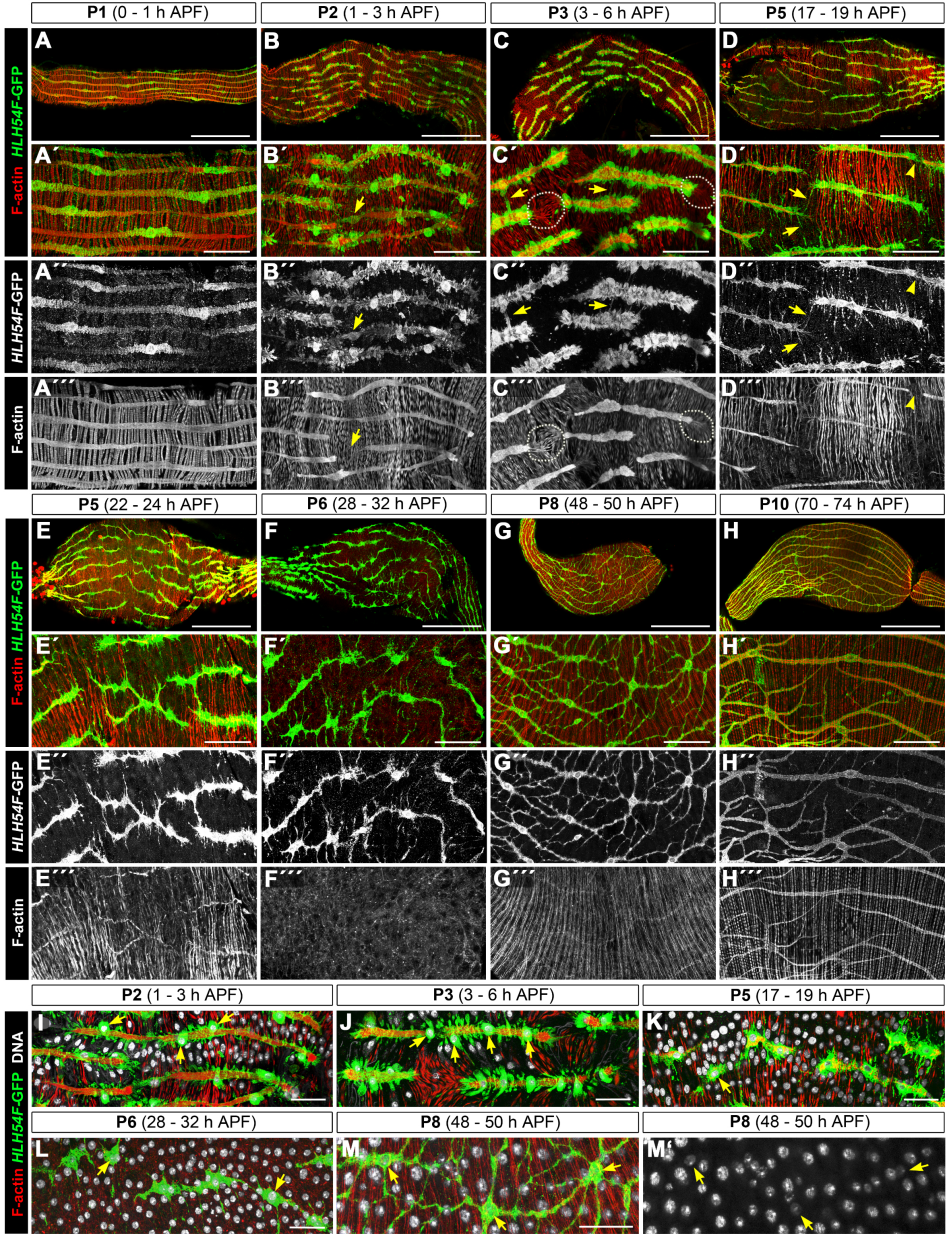
(A - H’’’) Dedifferentiation and redifferentiation of midgut muscles from pupal stage 1 (P1), 0 – 1 h after puparium formation (APF) until P10 (70 – 74 h APF). A – H show overviews of midguts (scale bars: 100 μm) and A’ to H’’’ show high magnification views (scale bars: 25 μm). At P1 (A – A’’’) midgut muscle morphologies appear normal but the longitudinal visceral muscles (LVMu’s) already lack striations. At P2 (B – B’’’), the phalloidin-labeled sarcomeric apparatus of LVMu’s begins to fragment but the GFP-labeled cytoplasm is still continuous (arrows). At P3 (C – C’’’), the LVMu’s are fragmented into short syncytia and form filopodial extensions (arrows) towards their neighbors. White circles denote interrupted LVMu areas with distorted arrangements of circular visceral muscles (CiVMu’s). In P5 (D – E’’’), increased filopodial extensions in LVMu’s (D, D’, arrows) and decreased striations in CiVMu’s are seen, and muscle fiber fragmentation continues (D – D’’’, arrow heads). At P6 (F – F’’’), some LVMu’s have fragmented into binucleated or mononucleated cells, lack F-actin, and the CiVMu’s show maximal dedifferentiation with absent F-actin as well. At P8 (G – G’’’), only mononucleated LVMu cell bodies are seen that form an extensive network of delicate cellular extensions. CiVMu’s and LVMu’s show reappearance of F-actin but lack of striations. At P10 (H – H’’’), LVMu’s have become syncytial again and have largely aligned in parallel, except in some areas where the filopodial network is still present. F-actin filaments begin to form striations. (I – M’) show high magnification views of midguts with additional nuclear Hoechst staining (white) to distinguish syncytial vs. mononucleated cells (scale bars: 15 μm). In P2 and P3 (I, J), the nucleocytoplasmic islands (arrows) of LVMu’s jut out from the muscle fibers, which progressively fragment. From P5 to P8 (K – M’), increasing numbers of mononucleated LVMu myoblasts are present (arrows), which at P8 are connected through a network of cellular extensions.

## Description

In this experiment done contemporaneously with the study of Aghajanian et al. (2016) we re-examined the metamorphosis of the midgut musculature in more detail. Guts from staged animals carrying *HLH54Fb-eGFP* , which marks the longitudinal midgut muscles (Ismat et al. 2010; Reim et al. 2012), were isolated and stained with anti GFP and phalloidin to visualize F-actin. Within the first hour of pupariation (pupal stage P1), the longitudinal visceral muscles (LVMu’s) still show the larval pattern of parallel equidistant muscle fibers extending along the length of the midgut (Fig. 1A). The LVMu’s have largely lost their striations and GFP is distributed throughout their length, with higher concentrations being present in the cytoplasm near the muscle nuclei (Fig. 1A - A’’’). At this time, the circular visceral muscles retain their orthogonal arrangement of striated muscle fibers. However, shortly thereafter at stage P2 (1 - 3 h after puparium formation [APF]), significant changes become apparent in the morphology of both types of visceral muscles (Fig. 1B - B’’’, Fig. 1I). As already noted (Klapper 2000), the longitudinal visceral muscles start forming extensive lateral cytoplasmic protrusions along their entire length at this time. The larger protrusions include the nuclei and their adjacent cytoplasm, whereas smaller protrusions are formed in the intervals between different nuclei. Isolated clumps of GFP between muscle fibers suggest that some of the cytoplasmic material from these protrusions is being shed (Klapper 2000). The phalloidin staining shows at some positions thinning and at others interruptions of the actomyosin arrays, and the remaining F-actin filaments lack striations (Fig. 1B’-B’’’, Fig. 1I). Phalloidin signals are notably increased at the newly generated ends of the sarcomeric arrays, indicating that F-actin may have retracted from the point of rupture and therefore is present at higher density (Fig. 1B’’’). While some of the F-actin gaps are still bridged by GFP-stained cytoplasm (Fig. 1B’’, B’’’, arrow), at other positions both the F-actin and the GFP signals are interrupted, which indicates the formation of complete fissures of the muscle fibers. The circular fibers are still striated at P2 but the striations of individual myofilaments are no longer in register (Fig. 1I). In addition, the circular fibers become thinner and packed more densely, which correlates with the progressive shortening of the midgut. At 3 - 6 h APF (pupal stage P3), the fragmentation of longitudinal fibers has progressed and each of the resulting pieces typically contains 3 - 6 nuclei at this time (Fig. 1C - C’’’, Fig. 1J). The majority of their cytoplasmic protrusions have become more pronounced but in addition, fine filopodia-like protrusions connect neighboring muscle fragments (Fig. 1C’, C’’, arrows). With phalloidin, thin F-actin filaments are seen that extend from the ends of the muscle fragments. These appear to belong to thin protrusions that attach to circular muscle fibers and pull at them, as indicated by the chevron-shaped bents of the circular fibers in the areas between the ends of longitudinal fibers (Fig. 1C’’’, circled; Fig. 1J). During early stage P5 (17 - 19 h APF), the longitudinal muscle fragments and particularly their F-actin arrays become thinner. Their cytoplasmic protrusions become long and thin, often contacting the corresponding protrusions emanating from their neighboring muscle fragments that lie in parallel (Fig. 1D - D’’’, arrows). Muscle fragmentation progresses during this stage, which is evident from the appearance of additional thin stretches with reduced or absent F-actin (Fig. 1D’ - D’’’, arrow head) and the presence of smaller fragments with only two nuclei or one nucleus in them (Fig. 1K, arrow). Similar arrangements are observed during late P5 (22-24 h APF; Fig. 1E - E’’’), when the F-actin arrays of the longitudinal fibers have deteriorated further and become extremely thin. Between P3 and P5, the midgut continues to shorten and the resulting lengthening of the circular fibers could partially explain their observed thinning and crowding that progresses during these stages. In addition, during P5, the striations of the circular fibers become less distinct and F-actin staining diminishes (Fig. 1E’’’; Fig. 1K). At P6 (28 - 32 h APF), the dedifferentiation of the midgut visceral muscles has reached its peak and neither the cells derived from the longitudinal muscles nor those derived from the circular muscles can be stained for F-actin any longer (Fig. 1F’’’) (Klapper 2000; Aghajanian et al. 2016). Many of the longitudinal visceral muscle derived cells appear mononuclear or binuclear and contact their neighboring cells through thin protrusions (Fig. 1F - F’’; Fig. 1L). However we cannot rule out completely that some of these delicate extensions that link cells along the anterior-posterior axis still provide tenuous cytoplasmic connections. At stage P8 (48 - 50 h APF), when the midgut has shortened maximally, the longitudinal muscle derived cells form an extensive network of fine protrusions that form numerous contacts with one another (Fig. 1G - G’’; Fig. 1M). In these cells, weak F-actin signals can be seen again, particularly within their more prominent connections, and the paths of these F-actin fibrils most likely predict which of the connections will mature into the adult longitudinal muscles (Fig. 1G’’’). In the circular muscles, myofibrils have also been reconstituted, although at this time there appear to exist only two fibrils per muscle, which still lack clear striations (Fig. 1G’, M). At stage P10 (70 - 74 h APF), the newly formed adult longitudinal midgut muscles can be discerned more clearly. Particularly in the posterior portion of the midgut, most of them are arranged in parallel again, whereas in the anterior portion there are still numerous branches and cellular extensions that contain F-actin (Fig. 1H - H’’’). The circular muscles now display distinct striations, although the striations of individual myofibers are not yet aligned in register (Fig. 1H’’’). By ~80 h APF, all branches and protrusions of the longitudinal muscles have been eliminated and the midgut muscles have formed the regular orthogonal arrangement of longitudinal and circular striated muscle fibers as seen in the adults (data not presented).

Our observations show additional features to those presented by Klapper (2000) and Aghajanian et al. (2016), and yield additional information about these dedifferentiation/redifferentiation events. Our results agree with the proposition by Aghajanian et al. (2016) that longitudinal visceral muscles in *Drosophila* do not persist as such during metamorphosis, although their nuclei with some surrounding cellular contents do persist and rebuilt the adult muscles, which can explain previously reported observations (Klapper 2000; Klapper et al. 2001). Similar events of dedifferentiation and fragmentation of striated muscles, and their redifferentiation after renewed myoblast fusion, were described for specific segmental alary muscles of the heart during *Drosophila* metamorphosis (Schaub et al. 2015). However, as described herein and by Aghajanian et al. (2016), the myoblasts derived from longitudinal visceral muscles fuse exclusively with one another to re-establish essentially the same type of muscle in the adult, whereas the alary muscle derived myoblasts recruit additional fusion partners from the pool of adult muscle precursors and transdifferentiate into a different type of muscle, namely the ventral longitudinal muscles of the adult heart (Schaub et al. 2015).

## Methods

*Drosophila HLH54Fb-eGFP* was generated by cloning the HLH54Fb enhancer element into P{GreenH-Pelican} and subsequent random genomic P-insertion (Barolo et al. 2000; Ismat et al. 2010). For gut preparations from pupae at 0 – 12 h and after 78 h APF, the pupae were attached anteriorly with a tungsten needle, opened at the posterior end with micro-scissors, cut and pried open along the dorsal midline, and the gut was removed with a forceps. For 12 – 78 old pupae pinned down at their shell anteriorly, the posterior end was cut open and the pupal contents including the gut were squeezed out with a forceps. After rinsing the contents with a pipet the gut was transferred into the fixing solution. Fixation was for 20 – 40 min in PBS, 0.1% Tween 80 containing 3.7% formaldehyde. Guts were stained over night at 4°C with Phalloidin-Atto-550 (1:3000; Sigma-Aldrich #19083) and mouse anti-GFP (1:100; Invitrogen, now ThermoFisher Scientific #MA5-15256) or rabbit anti-GFP (1:2000; Invitrogen, now ThermoFisher Scientific #A-11122), and Hoechst (10 μg/ml; Sigma-Aldrich #63493), 2 h at 25 **^°^** C with secondary antibodies (DyLight 488-conjugated, 1:200; Invitrogen, ThermoFisher Scientific #35502 or #35552) (with appropriate PBS, 0.1% Tween washes) and embedded in Vectashield (Vector Laboratories #H-1000-10). Images were acquired on a Leica SP5II confocal laser scanning microscope (HC PL APO20x/0.70 and HCX PL APO 63x/1.3 objectives, both with glycerol) using the LAS AF (Leica) software. Pupal stages are defined as previously described (Bainbridge and Bownes 1981).
